# Improving the Quality of Care in Care Homes Using the Quality Improvement Collaborative Approach: Lessons Learnt from Six Projects Conducted in the UK and The Netherlands

**DOI:** 10.3390/ijerph17207601

**Published:** 2020-10-19

**Authors:** Reena Devi, Graham Martin, Jay Banerjee, Louise Butler, Tim Pattison, Lesley Cruickshank, Caroline Maries-Tillott, Tracie Wilson, Sarah Damery, Julienne Meyer, Antonius Poot, Peter Chamberlain, Debbie Harvey, Clarissa Giebel, Kathryn Hinsliff-Smith, Neil Chadborn, Adam Lee Gordon

**Affiliations:** 1 School of Healthcare, University of Leeds, Leeds LS2 9JT, UK; 2 The Healthcare Improvement Studies Institute, University of Cambridge, Cambridge CB2 0AH, UK; Graham.Martin@thisinstitute.cam.ac.uk; 3 University Hospitals of Leicester NHS Trust, Leicester LE1 5WW, UK; jb234@leicester.ac.uk; 4 Department of Health Sciences, University of Leicester, Leicester LE1 7RH, UK; 5 Department of Ageing and Complex Medicine, Salford Royal NHS Foundation Trust, Salford M6 8HD, UK; Louise.Butler@srft.nhs.uk (L.B.); Tim.Pattison@srft.nhs.uk (T.P.); 6 Essex County Council, Chelmsford CM1 1QH, UK; Lesley.cruickshank@essex.gov.uk; 7 West Midlands Academic Health Science Network, Birmingham B15 2TH, UK; caroline.maries-tillott@wmahsn.org; 8 Walsall Clinical Commissioning Group, Walsall WS2 7JL, UK; tracie.wilson1@nhs.net; 9 Institute of Applied Health Research, University of Birmingham, Birmingham B15 2TT, UK; S.L.Damery@bham.ac.uk; 10 School of Health Sciences, City University of London, London EC1V 0HB, UK; J.Meyer@city.ac.uk; 11 Department of Public Health and Primary Care, Leiden University Medical Centre, Hippocratespad 21, 2300 RC Leiden, The Netherlands; A.J.Poot@lumc.nl; 12 South Sefton Clinical Commissioning Group, Bootle L20 3DL, UK; Peter.Chamberlain@southseftonccg.nhs.uk (P.C.); Debbie.Harvey@sefton.nhs.uk (D.H.); 13 Department of Primary Care & Mental Health, University of Liverpool, Liverpool L69 3GL, UK; clarissa.giebel@liverpool.ac.uk; 14 National Institute for Health Research, Applied Research Collaborations, North West Coast, University of Liverpool, Liverpool L69 3GL, UK; 15 Faculty of Health and Life Sciences, De Montfort University, Leicester LE1 9BH, UK; kathryn.hinsliff-smith@dmu.ac.uk; 16 Division of Graduate Entry Medicine, University of Nottingham, Derby DE22 3NE, UK; Neil.Chadborn@nottingham.ac.uk (N.C.); Adam.Gordon@nottingham.ac.uk (A.L.G.); 17 NIHR Applied Research Collaboration–East Midlands (ARC-EM), University of Nottingham, Nottingham NG7 2TU, UK

**Keywords:** Quality Improvement Collaborative, Quality Improvement, Implementation Science, residential facilities, older people

## Abstract

The Breakthrough Series Quality Improvement Collaborative (QIC) initiative is a well-developed and widely used approach, but most of what we know about it has come from healthcare settings. In this article, those leading QICs to improve care in care homes provide detailed accounts of six QICs and share their learning of applying the QIC approach in the care home sector. Overall, five care home-specific lessons were learnt: (i) plan for the resources needed to support collaborative teams with collecting, processing, and interpreting data; (ii) create encouraging and safe working environments to help collaborative team members feel valued; (iii) recruit collaborative teams, QIC leads, and facilitators who have established relationships with care homes; (iv) regularly check project ideas are aligned with team members’ job roles, responsibilities, and priorities; and (v) work flexibly and accept that planned activities may need adapting as the project progresses. These insights are targeted at teams delivering QICs in care homes. These insights demonstrate the need to consider the care home context when applying improvement tools and techniques in this setting.

## 1. Introduction

The rising number of older people is a global phenomenon [1]. One option for older people who are not able to live independently is to live in a long-term care facility, such as a nursing home. An internationally agreed definition of nursing homes is provided by Sanford et al., defining these as facilities that “(i) provide 24-h functional support for people who require assistance with activities of daily living and have identified health needs, (ii) may or may not be staffed with health care professionals, (iii) provide long-term care and/or rehabilitation as part of hospital avoidance or to facilitate early hospital discharges (iv) do not function as a hospital ward and are not hospital based, and (v) may play a role in providing palliative and/or hospice care at end of life” [2]. There are differences between countries in the way that facilities operate, the way that care is financed, how quality of care is regulated, and in the mix and type of professionals employed [3]. For instance, nursing homes in the Netherlands employ a mix of health care professionals and care workers, and in the UK, nursing homes employ Registered Nurses and care workers (with wider healthcare input received from community services). On the other hand, residential homes (referred to as care homes in the Netherlands) in both the UK and the Netherlands employ care workers to provide direct care and healthcare professional input is received from community services. The general characteristics of residents living in nursing and residential homes, however, are similar [4]. In this article, we use the general term “care homes” to refer to both nursing and residential homes.

In the UK, the quality of care across the sector varies [5] and several initiatives dedicated to improving the quality and safety of care have been introduced in recent years. Since 2013, 15 regional Academic Health Science Networks (AHSN) across England have supported projects focused on improving quality and safety in care homes [6]. In 2016, National Health Service (NHS) England commissioned the Enhanced Health in Care Home Vanguards, an initiative to implement a suite of evidence-based interventions in care homes located in six areas of England [7]. In the Netherlands, national initiatives focused on improving quality of care in care homes were supported by the Dutch National Care for Frail Elderly Persons Programme, which took place from 2007 to 2016, and comprised a series of Quality Improvement (QI) initiatives and studies clustered around eight academic medical centres [8]. 

An approach used in several of these initiatives is the Quality Improvement Collaborative (QIC) intervention [9]. Various versions of the QIC intervention exist. One of the most prominent is the Breakthrough Series Collaborative, as developed by the Institute for Healthcare Improvement (IHI) [10,11]. A QIC based on the Breakthrough Series model is a multifaceted intervention that typically lasts 6–15 months [12] and generally includes five essential features: (1) a team of clinical and QI experts bring clinical and QI knowledge and lead the QIC; (2) local multi-professional teams take part and form the collaborative; (3) the collaborative focuses on a specific topic; (4) participants engage in structured activities; and (5) they use the IHI’s Model for Improvement to guide change [13]. The Model for Improvement is a framework used to guide improvement projects where goals are set and a process called the Plan–Do–Study–Act (PDSA) cycle is used to test the impact of changes [14]. A PDSA cycle is a cyclical process of planning change (plan), actioning plans (do), observing and reflecting on the result (study), and modifying plans to address what has been learnt (act) [14]. Previously, collaboratives have been studied mainly in hospital settings [9]. The recent use of QICs with care homes in the UK and the Netherlands provides an opportunity to examine and learn whether and how this approach works in this setting. 

A recent scoping review conducted by Chadborn et al. highlighted that while there is a body of evidence around QI strategies used in the care home setting, without detailed descriptions of how strategies are applied, the extent to which others can replicate and learn from them is limited [15]. The aims of this paper are firstly to provide detailed descriptions of six QIC projects carried out in care home settings in two countries, and secondly, to share insights and learning from these projects. 

## 2. Method

Representatives of teams delivering QIC interventions were identified through our networks and UK national organisations including the British Geriatrics Society, AHSNs, the Health Foundation Q Network, and Health Services Research UK. Representatives attended an initial face-to-face meeting where detailed descriptions of each QIC intervention were presented and structured using the Template for Intervention Description and Replication (TIDieR) standardised reporting template [16]. This was followed by a series of face-to-face, electronic, and telephone meetings where the TIDieR framework descriptions were used to elicit discussion about lessons learned during conduct of the QICs. The focus was to find lessons that applied across more than one QIC initiative and those specific to the care home setting, as opposed to generic lessons that might apply to QICs conducted in other contexts. A list of lessons learnt is provided and each is summarised, outlining the challenges faced and the ways that these were addressed. The learning is targeted at teams leading and facilitating QICs in care homes.

## 3. Results

### 3.1. QICs in the Care Home Setting

The insights in this article are based on six QIC projects. Five were conducted in the UK and one in the Netherlands:

1. The Proactive Healthcare of Older People in Care Homes collaborative (PEACH);
2. Safer Care Homes;
3. Promoting Safer Provision of Care for Elderly Residents Collaborative (PROSPER);
4. The Safer Provision and Caring Excellence (SPACE) Programme;
5. The Medical care Optimalisation Care home Implementation (Medische zorg Optimalisatie Verzorgingshuizen Implementatie Traject)—MOVIT project;
6. The South Sefton Care Home Innovation Programme (CHIP).

Projects took place in five areas of England (Nottinghamshire, Salford, Essex, Walsall and Wolverhampton, and Bootle) and one area in the Netherlands (Leiden). The earliest project started in 2009 (MOVIT), the most recent started in 2017 (Safer Care Homes), and the length of completed programmes ranged from 13 months (Safer Care Homes) to 42 months (MOVIT), with one project still ongoing since 2014 (PROSPER). Projects’ specific aims varied. The PROSPER, Safer Care Homes, and SPACE QICs focused on improving safety and reducing avoidable harms. The PEACH QIC aimed to improve healthcare and used Comprehensive Geriatric Assessment (CGA) as a template to guide discussions. The MOVIT project aimed to improve fragmentation of medical care and the CHIP QIC focused on reducing ambulance conveyances. 

### 3.2. Descriptions of the Quality Improvement Collaborative Initiatives

Detailed descriptions of each QIC are provided in Table 1, Table 2, Table 3, Table 4, Table 5 and Table 6, broadly following the TIDieR template, with the addition of information on evaluation activities undertaken for each project.

### 3.3. Delivering a QIC in the Care Home Sector: Lessons Learnt

Five “lessons learnt”, specific to the care home sector and observed across more than one QIC initiative, were identified. These are listed in Box 1 and summarised below.

Box 1 **Applying the QIC approach in the care home sector: what have we learnt?**

1. Data are not always readily available in the care home sector; thus, sufficient resources are needed to support collaborative teams with:
  - Collecting the data needed to test the impact of change. Data collection burden could be reduced by identifying ways that data collection might be incorporated into care home routine practice in an intuitive way (e.g., the Falls Safety Cross approach).
  - Processing and interpreting data. Ensure data are presented in an accessible way, particularly for those who have not previously used data to evaluate change.
2. Make a conscious effort to create an encouraging and safe environment where collaborative members feel valued, connections are built in and across collaborative teams, and any perceived hierarchies between care home and healthcare staff are minimised. The following techniques help: (i) use appreciative language, (ii) celebrate achievement, (iii) facilitate ice-breaker activities, (iv) set ground rules, (v) reimburse care home staff time, and (vi) carry out small gestures, e.g., high quality catering at collaborative shared learning events.
3. Recruit collaborative teams and QIC lead/facilitators who have established and longstanding relationships, as these relationships are particularly important in enabling faster progress with QI in care homes.
4. People living in care homes receive input from multiple professionals employed across a mix of organisations. For this reason, lines of responsibility may be unclear, and there may be differences in what is considered a priority. Regularly check project ideas are agreed by team members to be: (i) within their job role and responsibility and (ii) a local priority.
5. The use of QICs in the care home setting has not been widely described and understanding around the types of activity that work well and which do not is limited. As we continue to generate learning in this area, it is important to work flexibly, accepting that activities may not go as planned and modifying planned activities if needed.

### 3.4. Plan for the Resource Needed to Support Collaborative Teams with Collecting, Processing, and Interpreting Data

Collaborative teams taking part in a QIC carry out projects where changes are made that aim to improve the quality of care, and PDSA cycles are used to test the impact of those changes. Data are an essential ingredient in assessing whether or not changes result in improvement. However, the data to inform PDSA cycles are not readily available in care homes in the same way as they are in health sector settings (Box 1, point 1a). Or, if data are available, the specific nature of the data might not match the specific aims of the QI projects. For example, collaborative teams in the Safer Care Homes collaborative faced challenges with establishing a baseline number of falls with harm and medication errors in care homes, and for this reason, the QIC facilitators worked closely with care homes to support data collection. Indeed, QIC facilitators were needed to provide support in all projects assessed in this paper. Collaborative team members provided data and the QIC facilitators then processed the data, constructing data dashboards and runtime charts. In some cases, QIC facilitators also helped with collecting data. In addition to this, the frontline staff taking part in the collaboratives may not have worked in this way before, where changes to care are made and data are used to evaluate the impact, and for this reason, QIC facilitators helped with interpreting data and reviewing PDSA cycles, arranging meetings where the data were interpreted and discussed.

A shared observation across projects was the importance of presenting data in an easy-to-digest way that enabled collaborative teams to review the impact of their changes (Box 1, point 1b). The level of support with data collection, processing, and interpretation required substantial resources from the QIC facilitators, which was not always anticipated during the planning phases of projects. To reduce data collection burden, the SPACE and PROSPER projects looked for ways that data could be collected within care home routine practice in an intuitive way. Both the SPACE and PROSPER projects used the Falls Safety Cross (for example, see https://www.livingwellessex.org/media/571058/falls-safety-cross.pdf), a data collection tool where care workers indicated the number of falls per resident on a prominent visual aide memoire display. This allowed falls-related data to be collated over time and allowed the data to be used to link to the improvement aim. Over time, care homes modified the Falls Safety Cross to also capture additional aspects of care quality such as incidents of challenging behaviour or resident hydration. An additional benefit for the care home was that the collection of data provided evidence of their safety culture, which was noted positively during inspections by the English regulator, the CQC.

### 3.5. Create Encouraging and Safe Working Environments

Care homes are heavily regulated, face negative public perceptions and stigma, and the majority of care homes are run by private companies (in the UK), and thus, there can be a sense of competitiveness between care home organisations. For these reasons, those working in care homes might be wary of and have reservations towards both those external to the care home sector (for example, academic researchers and those working in an NHS or commissioning role) and those from other care home organisations.

Across all QICs projects, conscious efforts were made to help create environments where participants felt safe and valued (Box 1, point 2). One technique was the use of appreciative language when asking collaborative teams for project progress updates. This could involve, for example, asking teams to focus on “*What worked well and why?*”, “*How would you want things to be?*”, “*How can we work together to make this happen?*”, and “*What needs to be in place to make it happen more of the time?*”. Phrasing questions carefully using appreciative language helped to focus on moving forward instead of focusing on barriers or problems. Another technique used by QIC facilitators was to create a celebratory atmosphere during shared learning events by congratulating collaborative teams, sharing positive stories. Ice-breaker activities helped to create an atmosphere of inclusivity and encourage connections amongst collaborative teams. Establishing agreed ways of working (e.g., listen to whoever is speaking, no question is a silly question, do not speak using acronyms) helped to create a safe environment and reduce perceived hierarchical imbalances, particularly where teams were mixed in seniority and/or professional status. In some projects, backfill payments were provided to care home staff to reimburse the cost of the time taken to attend meetings and help with arranging staff cover. Small gestures also helped to create an atmosphere where collaborative members’ attendance and input was valued, such as providing high-quality catering at collaborative shared learning events. An observation across all projects was that over time, trust, relationships, and a sense of community developed where care homes started to work more collaboratively, openly sharing their ideas and learning and resources (e.g., training resources).

### 3.6. Seek Out Collaborative Teams and Leads/Facilitators with Existing and Longstanding Relationships

A shared observation across QIC projects was the time needed to establish teams, build trusting relationships, and develop and implement improvement projects should not be underestimated (Box 1, point 3). The MOVIT project’s experience suggests that recruiting and forming collaborative teams takes at least one year, establishing team rapport and developing QI projects could take up to six months, and depending on the improvement projects, the time required to be able to notice effects could be a matter of years. The PEACH study recognised this and actively sought out collaborative teams where there were established relationships, enabling teams to “hit the ground running”. Similarly, good working relationships between the collaborative members and the QIC leadership team also help with project progress. The Safer Care Homes project leads used their pre-existing relationships and recruited care homes known to the QIC leads and facilitators, and found faster progress where collaborative team members knew the facilitating staff. In projects where the QIC project facilitators were not known to the care homes taking part, it was found that progress became easier once trust was established and any previous disputes or misunderstandings resolved.

### 3.7. Clarify Collaborative Member Priorities and Lines of Responsibilities

The care home sector is distinctive in that there are multiple organisations and multiple and different health or social care professionals provide health- and care-related services to residents. When delivering a QIC project, those leading and facilitating need to ensure collaborative teams develop QI projects which are directly related to team member job roles and responsibilities, and in which team members believe their job role and responsibilities could have some influence (Box 1, point 4). For example, the Safer Care Homes project set out to reduce falls, pressure ulcers, and medication errors. In the initial stages, care home staff viewed the cause of these issues as external to the home, believing that pressure area damage was acquired during hospital admissions and not inside the care home. In this case, QIC leads sought to discuss the factors which affected resident safety both inside and outside the homes, and participants started to engage when they saw they had some influence. The variety and mix of health and social care professionals may also mean differences in perceived priorities. In a similar way, it is worth spending time checking collaborative teams are invested and view QI project topics as a priority. For example, in the MOVIT project, collaborative teams spent some time at the beginning of the project reflecting on and choosing project ideas that aligned with their priorities. This ensured collaborative teams worked on topics that mattered to them. Allowing teams to work on their local priorities helped to maintain the ownership and buy-in needed to implement change. Working in this way and allowing local priorities to take precedence might not be possible if projects are funded to achieve objectives focused on a predefined topic.

### 3.8. Work Flexibly and Modify Planned Activities Where Needed

The experience shared across projects is that whilst QIC facilitators may have had project activities planned, they often had to work flexibly and adapt activities in response to collaborative teams, adapting their activity plans as they went along (Box 1, point 5). This is true in all QICs, but particularly when working in care homes because processes and principles which work for community healthcare or hospital teams will need adaptation to work in this setting. For example, the CHIP project reduced original meeting durations to enable greater focus and maximum attendance, and the PEACH project changed the programme remit from one around Comprehensive Geriatric Assessment, which members found difficult to understand, to one around delivering holistic care to residents. More examples around how QICs projects were modified are provided in Table 1, Table 2, Table 3, Table 4, Table 5 and Table 6. We suggest that project teams carry out initial pilot/set up phases. This would help to “test” planned activities, check feasibility, and examine potential modifications that might be needed. Initial pilot/set up phases would also help to build in the time needed to establish collaborative teams and build trusting relationships (Box 1, point 3).

## 4. Discussion

The extent of what we can learn from publicly available reports of QI in care homes is limited due to the lack of detailed reporting in this field [15]. This article helps to address this gap by providing detailed descriptions of how the QIC method has been applied and insight into the experiences of six projects using this methodology in care homes in the UK and the Netherlands. The insights described in this paper are also likely to be of value to those working in healthcare settings. While there is a wide-ranging QI evidence base, there is also a wide-ranging care home evidence base, with limited interaction between the two. Currently, insightful learning from each literature base has not yet been brought together, and thus, insights which may surprise experts in QI may not surprise those who are expert in care homes, and vice versa. Bringing insights and learning together in one paper is an important step forward.

One common observation across projects was that QIC leads and facilitators had not anticipated the extent of support collaborative teams would need with collecting, processing, and interpreting data. Use of baseline data and comparison groups to determine the effect of changes made to practice is rare in the care home sector, but of great importance to robust evaluation. It is important to take time establishing the data needed at the beginning of the project so its implementation and impacts can be properly monitored. The observation around data collection is perhaps unsurprising in countries where care home sector data are not routinely available and are held across different organisations. The collaborative nature of the QIC approach, though, could bring together key stakeholders from across organisations where data are held, and thus, help with accessing relevant data. This is an issue in countries as diverse as England, Austria, Portugal, and Brazil. In England, numerous ongoing research studies are focused on addressing this [25,26]. Countries such as the Netherlands and the United States have more consistent approaches to collecting care home quality benchmarking data [27,28].

Our other observations provide practical recommendations that are consistent with, and build upon, the wider care home literature. Previous findings show when dialogue with care homes is appreciative and focused on what is working well, this helps to develop practice in care homes [29]. Evidence also shows that working relationships in the care home sector are of particular importance, as successful innovations in care homes are established on a foundation of longstanding collaboration and trust [30]. In addition, previous evidence highlights how the lines of responsibility for those working in and with care homes are not always clear, as people living in care homes receive care from professionals working in different organisations. Thus, there can be uncertainty and dispute over roles and responsibilities for particular aspects of care [31].

### Strengths and Limitations

A key limitation is that the learning described here reflects the experience and perspectives of those who led and facilitated QICs, and not the views of collaborative participants. The insights we present were developed through a relatively unstructured, discussion-based approach, though our use of the TIDieR framework enabled us to identify, present, and compare key points of similarity and difference across the cases. Some of our observations might be unsurprising to those working in care homes; however, we believe these care home-specific insights may not be fully appreciated by improvement practitioners who work outside the care home setting.

The main strength of our article is that it addresses a gap in the existing QI and care home evidence base. A recent review of QI strategies applied in care home settings included 65 studies, and reported that to date, the evidence in this field lacks comprehensive reporting, limiting the extent to which others can replicate and learn from existing work [15]. This paper makes a start in addressing this gap. To our knowledge, this article is the first to provide detailed descriptions of multiple QICs applied in the care home setting and describe learning from across these projects. Our detailed descriptions are structured using standardised reporting (the Template for Intervention Description and Replication—TIDieR). Reporting templates have not yet been used in the existing evidence base. Nevertheless, we have only begun to scratch the surface of learning from collaborative projects in care homes. We recommend that future research builds on this foundation by continuing to comprehensively describe how QICs are applied in this setting and conducting in-depth process evaluations to generate more learning about how to apply QIC methodologies in the care home sector.

## 5. Conclusions

As Marshall et al. put it, “frontline practice is messy, it is never possible to do things perfectly, and good improvers are always learning” [19]. The experiences described here illustrate that improvement tools and techniques cannot to be taken “off-the-shelf” and applied without adaptation to the local context [19]. Our detailed descriptions of how the QIC approach has been applied in care homes, and the practical lessons learnt, will enable future teams to progress more quickly. We recommend that teams leading QICs in this sector continue to share detailed descriptions, given the paucity of literature available on the topic to date.

## Figures and Tables

**Table 1:** Description of the PEACH Quality Improvement Collaborative.

Brief Name	The PEACH Collaborative
Why	The aim was to improve healthcare for care home residents, and CGA was used to guide discussions.
Where	Nottinghamshire, UK. Collaborative shared learning events were carried out at a university location, and in between events (action periods), teams met in local care homes and at local Clinical Commissioning Group (organisations which plan and purchase healthcare services) locations.
Who provided	The PEACH collaborative was delivered by a team comprising a locally known clinical academic geriatrician, a nurse leader with expertise in appreciative inquiry to promote quality of life in care homes, a Health Foundation QI Fellow, and a researcher with interest in improvement science. The overall PEACH programme was funded by The Dunhill Medical Trust (grant number FOP1/0115). The collaborative shared learning events were funded by the East Midlands AHSN Patient Safety Collaborative (https://www.emahsn.org.uk/our-work/patient-safety).
Recipients	The collaborative took place across a region which has four distinct sites, and a team formed in each site. In each site, the person responsible for planning and purchasing healthcare services (commonly referred to as “commissioners” in the UK) for older people recruited a team. Teams were multidisciplinary and included general practitioners (GP), nurses, therapists, geriatricians, pharmacists, dementia specialists, care coordinators, care home workers/managers, and voluntary sector staff. Members of the public with experience of care homes were also recruited to teams. The configuration of teams varied and depended on local resource and staff availability.
How	Face-to-face meetings.
When and how much	18 months (September 2016 to February 2018), with four collaborative shared learning events that took place approximately every 6 months.
What (materials and procedures)	Collaborative shared learning events: The events included: Allocated time for teams to discuss and reflect on their local needs and priorities. Allocated time for teams to brainstorm and develop QI plans. Sessions for each team to present and share their project ideas, progress, and experiences of the improvement journey, describing challenges, successes, and lessons learnt around how to overcome barriers. Educational/learning sessions (described below). Networking opportunities. Educational/learning sessions: the events included educational elements, with training delivered on: QI techniques: setting SMART (Specific, Measurable, Achievable, Realistic, Timebound) objectives and testing change ideas using a PDSA approach. An educational game using “Mr Potato Head” was carried out to demonstrate the PDSA approach, teaching teams how to set goals, and test change ideas CGA and using this approach to care for older people. Action period group meetings: during action periods (the time in between each shared learning event), teams met at their own site locations to review and progress their improvement projects. Coaching: a Health Foundation-trained QI fellow on the team (JB) provided coaching and mentoring to individual teams, both at shared learning events and also during the action periods. Signposting teams to relevant contacts and resources: when collaborative teams faced challenges, the improvement team helped by signposting to relevant contacts and resources. Newsletter: provided project updates (i.e., meeting dates) and team stories describing progress with QI projects. Shared through email, with approximately three newsletters per year. Administrative support: the project improvement team provided the collaborative teams with administration support during action periods, for example, arranging meetings and circulating meeting agendas/minutes. Support with data collection: the collaborative intervention was one component of a programme of work which included work packages orientated around evaluating the activity of the QIC, collecting data around health care service use, and care home resident wellbeing. Collaborative teams were offered support with data collection and evaluation.
Tailoring	Shared learning events included features designed to create a safe working environment and reduce effects of perceived hierarchy amongst teams: Ice breaker activities to enhance relationship building. Time was spent at the beginning asking teams to consider items to add to a list of “ground rules”, for example, (i) no question is a silly question, (ii) everyone listen when someone is speaking, (iii) mobile phones on silent. Team members were asked to comply with these rules throughout the events. All activities maintained an appreciative enquiry approach, using positive and encouraging language, e.g., asking teams to focus on what is working well and why, envisaging how things could be, and identifying how to work together to make it happen. GPs and care home staff were provided with backfill payment for their time taken to attend events as they are independent sector workers and only able to attend meetings if adequate staff cover is arranged to cover workload.
Modifications to the programme	The original plans included carrying out conference calls as another way to meet and discuss progress with improvement work. The conference calls would take place during action periods and involve each collaborative team with the improvement team. One conference call was carried out and not repeated as face-to-face meetings were more effective for reviewing and discussing project progress.
How well	Over the course of the project 34 (out of 44) NHS and care home staff attended at least 2 (out of 4) collaborative meetings.
Project evaluation	Process evaluation to understand how the QIC approach works, for whom, and in what ways when used to implement and deliver CGA in care homes. This evaluation uses a realist methodology; a detailed research protocol is available elsewhere [17]. QI project evaluation to examine the impact on resident and service outcomes. A combination of interrupted times series, stepped wedge cluster design, and quasi experimental approaches were used, and are described in more detail by Usman et al. [18].

**Table 2:** Description of the Safer Care Homes Quality Improvement Collaborative.

Brief Name	Safer Care Homes
Why	The aim was to reduce medication errors, falls with harm, and pressure ulcers.
Where	Salford, UK. Collaborative shared learning events were held at a local centre for QI (http://www.haelo.org.uk/about-us/), and in between events (action periods), the collaborative met during peer exchange visits carried out at care home locations.
Who provided	The Safer Care Homes collaborative was delivered by a local organisation called Haelo: an innovation and improvement science centre based in Salford, and commissioned by Salford Clinical Commissioning Group. The Safer Care Homes collaborative was delivered by a team including an executive sponsor (Safer Salford board representative), a consultant geriatrician, a QI lead, a programme facilitator, and a data analyst (measurement support).
Recipients	Nine care homes (mix of residential and nursing) took part and collaborative members comprised care home managers and senior/junior care workers from each participating care home.
How	Face-to-face meetings.
When and how much	13 months (January 2017–January 2018), with four half-day collaborative shared learning events that took place quarterly, and monthly peer exchange visits.
What (materials and procedures)	In September 2016, a local expert panel met to set the aims of the Safer Care Homes collaborative. The panel included commissioners, general practitioners, community geriatricians, safeguarding leads, pharmacy leads, and care home representatives. A driver diagram was developed which set out the aims and objectives of the collaborative. Collaborative shared learning events included: Sessions for each care home to present and share their project ideas, progress, and experiences of the improvement journey, describing challenges, successes, and lessons learnt around how to overcome barriers. The improvement team presented analysed data from care homes to the whole collaborative. Allocated time for each care home to examine and reflect on data, and develop action plans. The improvement team encouraged care homes to generate and test ideas that were aimed at reducing falls, pressure ulcers, and medication errors. Educational sessions (described below). Networking opportunities. Educational sessions: each event included educational elements, with training delivered on QI methodology. Influence of the care home on harm reduction. Coaching, data collection, and project evaluation: members of the improvement team visited care homes weekly to provide additional support with QI training and provided each home with data dashboards constructed from data submitted from the home. Peer support and exchange visits: collaborative members visited other care homes part of the collaborative as another way to share and exchange knowledge and experiences. This helped to develop a support network between the care homes. Awards and celebrating good work: at the summit event, care home members were recognised for their achievements with awards. All received an award for completing the programme, with additional awards agreed by the improvement team for “most improved”, “most innovative PDSA”, and “best use of improvement methodology”.
Tailoring	After the programme was completed, the improvement team adapted the model for improvement for a care home audience. This is called the “six steps to improvement” and is based on the learning and feedback from participants. This is available online at: https://safersalford.org/wp-content/uploads/2018/07/6-steps-to-improvement-30.04.18.pdf.
Modifications to the programme	Establishing a baseline number of falls with harm and medication errors was difficult, and for this reason, the improvement team worked closely with care homes to provide support with data collection and analysis. Initially, the improvement team planned that care homes would come up with their own innovative change ideas to test; however, the care homes preferred the improvement team to provide ideas based on evidence. One example of a change idea used to improve rate of falls is “pimp my zimmer”, an intervention where resident walking aids are personalised and decorated to help residents recognise and use their own walking aid, and also allow staff to recognise when a resident is using the incorrect walking aid (https://safersalford.org/case-study-pimp-my-zimmer/). Part-way through the collaborative period, it was recognised that care homes valued time to share and learn from one another and so “peer exchange visits” (exchange visits hosted in participating care homes) were introduced to enhance shared learning, exchange ideas, and develop support networks. Education and training on the influence of care home on harm reduction was introduced to help care homes see they can influence the reduction in harm, e.g., changing the belief that falls were either inevitable or caused by factors external to the homes. Although the focus of the collaborative was to reduce falls, pressure ulcers, and medication errors, the majority of the homes focused on reducing falls during the collaborative. Focus on medication errors was introduced later during the collaborative. This occurred after one care home joined the collaborative part way through and showed an interest in this outcome. Following this, other care homes also started to show an interest in this outcome.
How well	Collaborative shared learning event attendance was not assessed.
Project evaluation	Success of individual change ideas was evaluated using data dashboards. Each care home was able to see the impact of each intervention, which informed ongoing tests of change. Improvement in QI knowledge was evaluated through a comparative improvement knowledge survey, performed at the start of the breakthrough series, after each shared learning event, and at the summit event. Qualitative data were collected to reflect the impact of shared learning and collaboration between each care home (https://safersalford.org/safer-care-homes-summit-2/).

**Table 3:** Description of the PROSPER Quality Improvement Collaborative.

Brief Name	PROSPER
Why	The aim is to reduce the number of harmful events (e.g., falls, pressure ulcers, and urinary tract infections) and improve the safety culture of teams.
Where	Essex, UK. Collaborative shared learning events were held in Chelmsford. Champion study days were held at five localities across Essex (Tendring, Colchester, Chelmsford, Basildon, and Harlow). Care home support visits were held at care home locations.
Who provided	The PROSPER collaborative was delivered by a team comprising a project manager and support officers with QI expertise (employed at Essex County Council), and community health practitioners with clinical expertise in falls, pressure ulcers, or urinary tract infections. The collaborative shared learning events were originally funded by the Health Foundation and have been sustained with Essex County Council and Better Care Fund funding.
Recipients	160 care homes (mix of residential and nursing) for older people, and 21 residential care homes for Learning Disability/Autism/Physical Sensory Impairment. The collaborative members comprised care home managers/deputies, senior/junior care workers, and domestic, kitchen, and maintenance staff from each participating care home.
How	Face-to-face meetings.
When and how much	An ongoing programme since 2014 consisting of two collaborative shared learning events per year, monthly care home support visits from members of the improvement team for the first 3 months (visits thereafter dependant on progress), and 10 champion study days a year.
What (materials and procedures)	Collaborative shared learning events: Teams present and share their project ideas, progress, and experiences of the improvement journey, describing challenges, successes, and lessons learnt around how to overcome barriers. Invited speakers deliver training (described below). Networking opportunities. Education and training: A PROSPER toolkit: paper and online (https://www.livingwellessex.org/quality/quality-innovation/prosper/prosper-toolkits/) resources to help care homes carry out quality improvement, comprising: SMART (Specific, Measurable, Achievable, Realistic, and Timebound) aim setting posters. Driver diagram templates to help collaborative teams explain what is needed to achieve goals (primary and secondary drivers). Worksheets to record small tests of change using the PDSA cycle approach. Data collection tools to measure the number of falls, newly acquired pressure ulcers, urinary tract infections, and hospital admissions each month. “Safety Cross”, a visual and colour coded data collection tool to display in care homes. The cross is split into days, and care staff use a colour code, using “green” for zero falls and “red” to indicate resident falls. An online mapping function allows care homes to input data online, and this then generates runtime charts to show progress over time. Tools for teams to carry out “Root Cause Analysis”. Invited speakers at collaborative shared events deliver training on relevant topics. Speakers included falls prevention specialists, occupational therapists, community health practitioners, community dental nurses, and continence/barrier cream suppliers. Speakers delivered training on pressure ulcers, falls, nutrition/hydration, infection control, catheter care, oral healthcare, manual handling and equipment, and urinary tract infections. Champion Study Days: intended for care staff who have taken the lead on implementing change in care homes. Domestics, kitchen, and maintenance staff also attended and received: Goody bags including: champion badge, double compact mirrors to check heels for pressure ulcers, keyrings, smoothie recipes, and toilet bowl sensor lights (Night my Light). Additional training on a range of subjects, e.g., falls prevention, infection control, nutrition/hydration, dementia, and pressure ulcers. Other training delivered in a hands-on and “fun” way. For example, making smoothies and frozen banana penguins to give staff ideas on boosting nutrition hydration for care home residents and using an ageing simulation suit during falls prevention training for staff to experience the physical aspects of frailty. Care home support visits: the improvement team visit care homes to enhance engagement and provide QI expertise and advice. Support with data collection and interpretation: monthly mapping runtime charts were provided to the homes from the improvement team based on data provided by the home. Runtime charts were discussed at care home support visits. Signposting: the improvement team helped by signposting care homes to relevant contacts, resources, and training. Monthly newsletter: sharing team progress, fun facts, and top tips (reinforcing methodology) through email and via the online care provider hub (https://www.livingwellessex.org/latest-news/prosper-newsletter/). Recognition of good practice: the PDSA worksheets, capturing care home QI activity, are provided as evidence to regulators who provide recognition in inspection reports.
Tailoring	The improvement team engage in ongoing discussions with collaborative participants and use their feedback to adapt tools. For example, data collection tools were modified with care home staff feedback to be simpler and less onerous to complete.
Modifications to the programme	Training around QI methodology included care home relevant examples, with driver diagrams and PDSA worksheet examples to help participants understand the concept of small changes. The community of practice events were adapted to allow more time for sharing of ideas and sharing care home experiences of their QI projects. More details around modifications made to the PROSPER intervention are reported by Marshall et al. [19].
How well	Collaborative shared learning event attendance was not assessed.
Project evaluation	Detailed reporting on the factors which helped/hindered the implementation of the PROSPER intervention components, changes made in care homes as a result (e.g., safety culture and safety processes), and resident and cost related outcomes are described in detail elsewhere by Marshall et al. [20] and UCL Partners Academic Health Science Partnership (https://www.livingwellessex.org/media/571025/prosper-final-evaluation-report.pdf).

**Table 4:** Description of the SPACE Quality Improvement Collaborative.

Brief Name	The SPACE Programme
Why	The aim was to promote a culture of continuous QI with potential to reduce avoidable harms in participating care homes.
Where	Walsall and Wolverhampton, West Midlands, UK. Mix of regional shared learning events organised by Walsall and Wolverhampton Clinical Commissioning Groups and delivery of training and ongoing support by SPACE programme facilitators in individual care homes.
Who provided	The intervention was delivered by two full-time facilitators (one in Walsall, one in Wolverhampton) with experience in QI. Appreciative Inquiry workshops to support positive safety culture were delivered by an external provider (https://www.appreciatingpeople.co.uk). The programme was funded by the West Midlands AHSN Patient Safety Collaborative (https://www.wmahsn.org).
Recipient	29 care homes: 11 nursing homes in Walsall (691 resident capacity) and 17 nursing homes and 1 residential home in Wolverhampton (1191 resident capacity). Collaborative members comprised care home managers, senior/junior nursing and care staff, staff in domestic, administrative, and maintenance roles, and activity coordinators.
How	Face-to-face meetings.
When and how much	24 months (December 2016 to December 2018) with eight half or full day collaborative shared learning events (four in Walsall and four in Wolverhampton). Monthly training in participating care homes attended by managers and staff, focusing on specific topics (described below). One to one coaching and support provided by facilitators throughout the programme (each home visited approx. weekly/fortnightly).
What (materials and procedures)	Collaborative shared learning events: Networking opportunities for attendees: exhibition stalls promoting resources related to harm free care (e.g., tissue viability) and stalls run by regional/national training providers (e.g., My Home Life, Skills for Care, Age UK). Skills development via group training and breakout sessions on harm-specific and general QI topics (e.g., PDSA cycles). Invited speakers gave overviews of national/regional challenges faced by the sector and facilitators presented on SPACE progress. Care homes presented QI projects, sharing success factors, barriers and how they were overcome, and sharing of their “improvement journey”. Education and training: training was delivered by the facilitators or by relevant specialist teams through small groups or large training workshops attended by staff from several care homes. Training delivered on: Leadership and culture: emphasised the importance of engaging stakeholders, leading/managing change, safety culture, and human factors training. Measurement for improvement: Model for Improvement Driver Diagrams were used to conceptualise QI and design projects. Based on SMART aims, choice and measurement of outcomes, and how improvement effectiveness can be tested using PDSA cycles. Communication and handover: focus on improving handovers between staff at shift change to support positive safety culture, e.g., developing safety boards to highlight key risks visually and minimise risk of errors and harms. Workforce development: training attendees asked to identify their learning from each session and describe how they would cascade that learning to colleagues once back at work to facilitate changes in care home practice. Support from facilitators: programme facilitators visited each home to provide ad hoc support and one-to-one QI coaching. This included reviewing PDSA data on specific QI projects and co-developing action plans, signposting towards relevant resources or external training opportunities, helping with data collection and interpretation, risk and harm monitoring, and providing regular data run charts to capture trends over time. Facilitators also supported homes to provide evidence to the Care Quality Commission (CQC) regulators, and address issues such as staffing and resistance to change. Recognition/sharing of best practice: Bi-monthly newsletters to highlight achievements, share learning, notify about forthcoming training events, and signpost to useful resources. Care home managers and staff also provided content (e.g., photos and articles describing events held at their home). Annual awards ceremony and “celebrating success” forum as part of the shared learning events, to recognise and reward innovative practice. Bi-monthly forums led by programme facilitators, attended by care home managers. Designed to build relationships, develop shared purpose, provide peer support, and share best practice. Programme sustainability: resource toolkit and best practice guidelines developed. Facilitator role in Wolverhampton integrated into the CCQ Quality Nurse Advisor (QNA) role, and quality assurance officers trained in QI. In Walsall, QI nurses undertake joint quality visits with the local authority.
Tailoring	Shared learning events carried out using Appreciative Inquiry principles, focusing on what works well and human factors to understand errors. Events included ice-breaker activities to enhance relationship building between teams and across care homes. Programme elements aligned with local and national priorities and best practice, e.g., CQC domains of care and hospital avoidance. Flexible design and delivery of care home-based training, integrating lessons learned from incidents, mapping exercises to encourage staff groups (maintenance, domestic etc.) to identify their own contribution towards particular aims. Training events designed to elicit a “commitment to act” from attendees and cascade learning to others to improve practice.
Modifications to the programme	Training flexibility: one-to-one coaching support with managers, small group training in the care home, larger workshop with staff from multiple care homes, and larger collaborative events to disseminate and share learning. Responsiveness: training was modified on an ongoing basis to respond to feedback and focused on topics identified as areas of interest. Adaptation: release of care home staff to attend training was challenging. Events were linked with specialist clinical training available from clinical partners, e.g., falls, tissue viability, and dementia. Underpinning theories: inclusion in year 2 of human factors principles, increased focus on oral care QI activity linked to chest infection reduction, and improving recognition and management of deteriorating care home residents. Co-design: emphasis on co-design of QI interventions between facilitators and managers/staff. Workforce development: workforce development and promoting opportunities for career advancement were offered.
How well	Collaborative shared learning event attendance was not assessed.
Project evaluation	Process and outcome evaluation was undertaken to assess programme design, implementation, and staff/service outcomes. Methods included care home manager and staff surveys, interviews with care home staff and key informants, quantitative analysis of pre and post implementation avoidable harms data, and observations of QI programme activities and training. The final SPACE evaluation is reported by Damery et al. [21].

**Table 5:** Description of the MOVIT Quality Improvement Collaborative.

Brief Name	The MOVIT Programme
Why	The aim was to reduce fragmentation of medical care and ensure care meets the increasing complex medical needs of residents.
Where	Leiden, the Netherlands. Regional meetings were held at a university location and teams also met locally in their care home locations.
Who provided	The MOVIT collaborative was led by a local general practitioner and team members included a professor of primary care at Leiden University Medical Centre, a project manager, a postdoctoral researcher with experience in geriatrics, and a liaison member of staff from a local GP organisation. The project was funded by the Dutch Ministry of Health via the National Programme on Elderly care.
Recipient	29 local teams were formed (serving 33 residential homes). Each comprised general practitioners, community pharmacists, elderly care physicians, and nursing home staff.
How	Face to face meetings.
When and how much	42 months (2009–2013) with 10 regional educational meetings that took place 2–3 times per year, and in between, regional teams met in their locations and received QI coaching.
What (materials and procedures)	Forming communities of practice: the regional project team actively identified and approached care providers of local residential homes (general practitioners, nursing staff, elderly care physicians, and pharmacists) and formed teams. Once formed, each team agreed a focus which reflected local needs around improving integrated care and translating this into an improvement plan. Collaborative shared learning events: Teams shared project ideas, progress, and experiences of the improvement journey, describing challenges, successes, and lessons learnt around how to overcome barriers. Allocated time for education sessions (described below). Networking opportunities. Educational sessions: Education sessions aimed to inspire teams and provide relevant clinical evidence-based knowledge. Sessions target improvement project topics and activities. Consensus and guideline development: the teams developed and implemented regional guidelines on a variety of topics: geriatric assessment, patient-based interdisciplinary meetings, medication management and distribution, wound treatment, and advanced care planning. Implementation and sustainability: Managers and governors of the organisations and financial and regulatory institutions were involved to consider future sustainability. Promoting sustainability by developing financial constructions for the participating professionals and organisations within regional and national frameworks. Newsletter: team success stories were shared using a project newsletter, shared through email, approximately every 6 months. Evaluation: the MOVIT project team included a research nurse who helped (to a limited degree) with collecting data to monitor and evaluate improvement project outcomes and study the QI process. QI coaching: each team received coaching from a GP trained in QI and with special interest in elderly care. There were approximately seven GPs providing coaching to teams. Coaches met regularly (every 6 months) to coordinate and exchange experiences.
Tailoring	The project team took a flexible approach, adapting and tailoring implementation activities to respond to the obstacles encountered.
Modifications to the programme	Government policy moved towards phasing out residential care during the project; as a result, collaborative teams adapted and worked on transporting care from the institutional context to that in the community. As a result, teams were expanded to include domestic and social care providers and related stakeholders.
How well	Collaborative shared learning event attendance was not assessed.
Project evaluation	A structured process description and analyses were performed to better understand the relation between the project activities, identify relevant contextual factors, and examine the fidelity and quality of the implementation [22]. General satisfaction and satisfaction with GP care were compared pre and post MOVIT implementation using a repeated cross-sectional study [23].

**Table 6:** Description of the South Sefton Care Home Innovation Program (CHIP) Quality Improvement Collaborative.

Brief Name	CHIP
Why	The aim was to reduce ambulance conveyances by 1/3 over 12 months from April 2015.
Where	Bootle, UK. The collaborative shared learning events were carried out at a neutral location (a hotel), and in between events (action periods), collaborative members continued to meet in their care home locations.
Who provided	The CHIP programme was led by two local general practitioners, and team members provided support with administration and with data collection and evaluation support. The project was funded by South Sefton Clinical Commissioning Group.
Recipient	31 care homes (both part residential and nursing homes) took part. The collaborative members comprised care home managers, senior and junior care staff, and over the course of the project, members from wider healthcare organisations provided input into improvement projects, such as community geriatricians, community matrons, pharmacists, palliative care specialists, voluntary organisations, tele-video equipment providers, and informatics.
How	Face to face meetings.
When and how much	36 months (April 2015–April 2018) with collaborative shared learning events every 2–3 months.
What (materials and procedures)	Forming the CHIP collaborative: prior to starting the CHIP collaborative, audits and interviews were carried out in individual care homes to understand and establish their needs. The CHIP collaborative was then designed to meet care home stakeholder requirements. Collaborative shared learning events: During events, each team was interviewed as a way of sharing progress, updates, and their experience of the improvement journey. Eduational and training (described below). Networking opportunities. Education and training: Training in QI methodology and QI techniques simplified through the use of games e.g., demonstrating PDSA cycles with Mr Potato Head. Training on how to use equipment being implemented in the care home, e.g., 24/7 tele-video in reach support. Training on basic observations and use of protocols with Edge Hill University. Awareness training from a variety of specialists. Care home teams were provided with support with data collection and interpretation: BI-level time series analysis was carried out and presented to care homes in an easy to digest way. Data were collected using data dashboards and monthly data trackers. Outcomes were focused on care outcomes and process measures at the care home level. Clinical support: Development of clinical protocols (e.g., standardised protocols topics such as falls and urinary tract infections). Relational coordination with care home matrons (care home matrons had easy and direct access to a community geriatrician, GPs, and other community specialist teams). Advanced care planning led by community matrons (the matrons collated background information, populated care plans, liaised with the GP or community geriatrician to complete and sign off care plans. Mostly done in liaison with GPs, and more complex cases referred to the community geriatrician). CHIP dashboard with “star” status: each home was given an individual attainment plan and “star chart” that helped them to reflect on areas of focus to enable scale up. CHIP champions: each care home selected a “CHIP” champion (e.g., care home manager or care staff). Champions functioned as a CHIP advocate and acted as the point of communication to both the improvement team and care home. Each champion was celebrated and recognised (e.g., given a badge). Care home support visits: the improvement team visited care homes regularly to carry out both reactive and proactive care. Any issues that they needed further support for were dealt with by contacting the GP or community geriatrician. Newsletters: monthly newsletter provided through email.
Tailoring	At the beginning, the improvement team spent time describing the purpose of the collaborative and their role, placing emphasis on the point that the improvement team were not inspecting or judging the care homes. Every collaborative shared learning event started with ice-breaker activities and a recap of the CHIP vision. Efforts were made to ensure meetings were facilitated in a way that created a safe, non-judgemental, positive, and celebratory atmosphere.
Modifications over the course of the programme	QI training materials were simplified as most of the collaborative members had no previous awareness of QI terminology or techniques (for example, simplified PDSA cycle templates were created). At collaborative learning events, instead of collaborative teams carrying out presentations, they were interviewed ‘on stage’ as a way of sharing progress to the collaborative. The time, day, and duration of collaborative shared learning events were changed to make it easier for collaborative teams to attend.
How well	On average, each collaborate shared learning event was attended by 63% of care homes.
Project evaluation	The impact of the CHIP collaborative on emergency calls and conveyances to hospital was evaluated using frequency analysis; more details are reported by Giebel et al. [24]. The CHIP project has been cited as an example of good practice by the CQC: see https://www.cqc.org.uk/sites/default/files/20160505%20CQC_EOLC_OVERVIEW_FINAL_3.pdf.
